# Breast and Contralateral Axillary Lymph Node Metastases After Ovarian Cancer Surgery: A Case Report

**DOI:** 10.1002/ccr3.73530

**Published:** 2026-09-17

**Authors:** Sihan Zhang, Zhiyuan Zhang, Jiaru Gao, Mengshen Wang, Minghui Zheng, Qiaonan Ye, Chenxi Yuan, Linjiao Jia, Wentao Li

**Affiliations:** ^1^ Department of Breast Surgery Henan Provincial People's Hospital, Zhengzhou University People's Hospital Zhengzhou China; ^2^ Department of Plastic and Burn Surgery, National Key Clinical Construction Specialty The Affiliated Hospital of Southwest Medical University Luzhou China; ^3^ Department of Computer Science and Engineering The Hong Kong University of Science and Technology Hongkong China; ^4^ Department of Pathology The Second Affiliated Hospital of Zhengzhou University Zhengzhou China; ^5^ Department of Plastic and Reconstructive Surgery Shanghai Ninth People's Hospital, Shanghai Jiao Tong University School of Medicine Shanghai China

**Keywords:** axillary lymph node metastasis, breast cancer, diagnosis, immunohistochemistry, ovarian cancer

## Abstract

Breast masses with contralateral axillary lymphadenopathy in patients with a previous ovarian tumor should not be presumed to be primary breast cancer. Careful review of prior pathology, broad immunohistochemical evaluation, and the least invasive tissue‐sampling strategy sufficient for diagnosis may help avoid misdiagnosis and unnecessary axillary surgery.

## Graphical abstract

Histopathological and immunohistochemical findings supporting ovarian‐origin metastasis in the left axillary lymph node. Hematoxylin and eosin staining and immunohistochemical staining supported metastatic ovarian carcinoma rather than primary breast cancer.

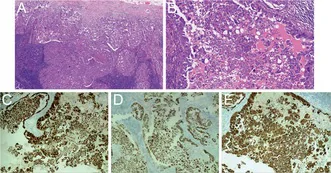

## 1 Introduction

Metastatic involvement of the breast from extramammary malignancies is uncommon and can closely mimic primary breast cancer on clinical examination and imaging [1]. Ovarian carcinoma is a rare source of breast metastasis, with only sporadic cases described in the literature [2, 3]. Breast metastasis accompanied by contralateral axillary lymph node involvement after surgery for a serous borderline ovarian tumor with focal microinvasion and negative pelvic lymph nodes, particularly when the recurrent lesions show a markedly higher histologic grade than the primary tumor, is even more unusual. We report such a case in a 62‐year‐old woman, highlighting practical diagnostic pitfalls and the importance of careful history taking, pathologic review, and targeted immunohistochemical evaluation.

## 2 Case History/Examination

A 62‐year‐old woman presented with a 2‐week history of a palpable right breast mass. Physical examination showed a firm, ill‐defined, poorly mobile, tender 1 × 1‐cm mass at the 5 o'clock position of the right breast, 1 cm from the nipple, and a hard, poorly mobile 3 × 2‐cm lymph node in the left axilla. No left breast mass or right axillary lymphadenopathy was detected.

She had no known family history of breast, ovarian, or other hereditary cancers, and no hereditary disease was documented in the available records.

The patient had undergone laparoscopic radical surgery for ovarian cancer, including omentectomy, on 3 November 2023 after ultrasonography and magnetic resonance imaging at an outside hospital had revealed a pelvic cystic mass. The routine postoperative pathology at the outside hospital reported a borderline serous cystadenoma with focal microinvasion (< 0.2 cm), positive peritoneal washing cytology, no pelvic lymph node metastasis (left pelvic nodes, 0/4; right pelvic nodes, 0/10), and no omental tumor involvement after omentectomy. According to the available clinical records, the primary ovarian tumor was recorded as International Federation of Gynecology and Obstetrics (FIGO) stage IC3. Immunohistochemistry showed CK7 (+), P53 (−), PAX‐8 (+), Napsin‐A (−), CDX2 (−), ER (partially +), P16 (+), CK20 (−), WT‐1 (+), and Ki‐67 (90%+). Postoperative chemotherapy for 3–6 cycles was recommended at the outside hospital; however, it was not initiated because the records documented poor postoperative physical tolerance. Detailed performance status or laboratory data explaining this decision were not available in the accessible outside hospital records. The main clinical timeline is summarized in Table 1.

**TABLE 1:** Timeline of the main clinical events.

Date	Clinical event
3 November 2023	Laparoscopic radical surgery for ovarian cancer, including omentectomy, was performed at an outside hospital; pathology showed a serous borderline tumor with focal microinvasion, positive washing cytology, negative pelvic nodes, and no omental tumor involvement
20 February 2024	Elevated serum cancer antigen 125; breast/axillary imaging and biopsies were performed
26 February 2024	Left axillary lymph node dissection and right breast mass excision were performed
4 March 2024	Positron emission tomography‐computed tomography (PET‐CT) showed multiple hypermetabolic lymph nodes suggestive of metastases
6 March 2024	Expert pathologic review confirmed serous borderline papillary cystadenoma with focal invasion
22 March, 12 April, and 8 May 2024	The first three cycles of paclitaxel plus carboplatin‐based chemotherapy were administered
13 March 2025	Computed tomography and tumor markers showed no radiologic disease progression

## 3 Differential Diagnosis, Investigations and Treatment

**FIGURE 1:**
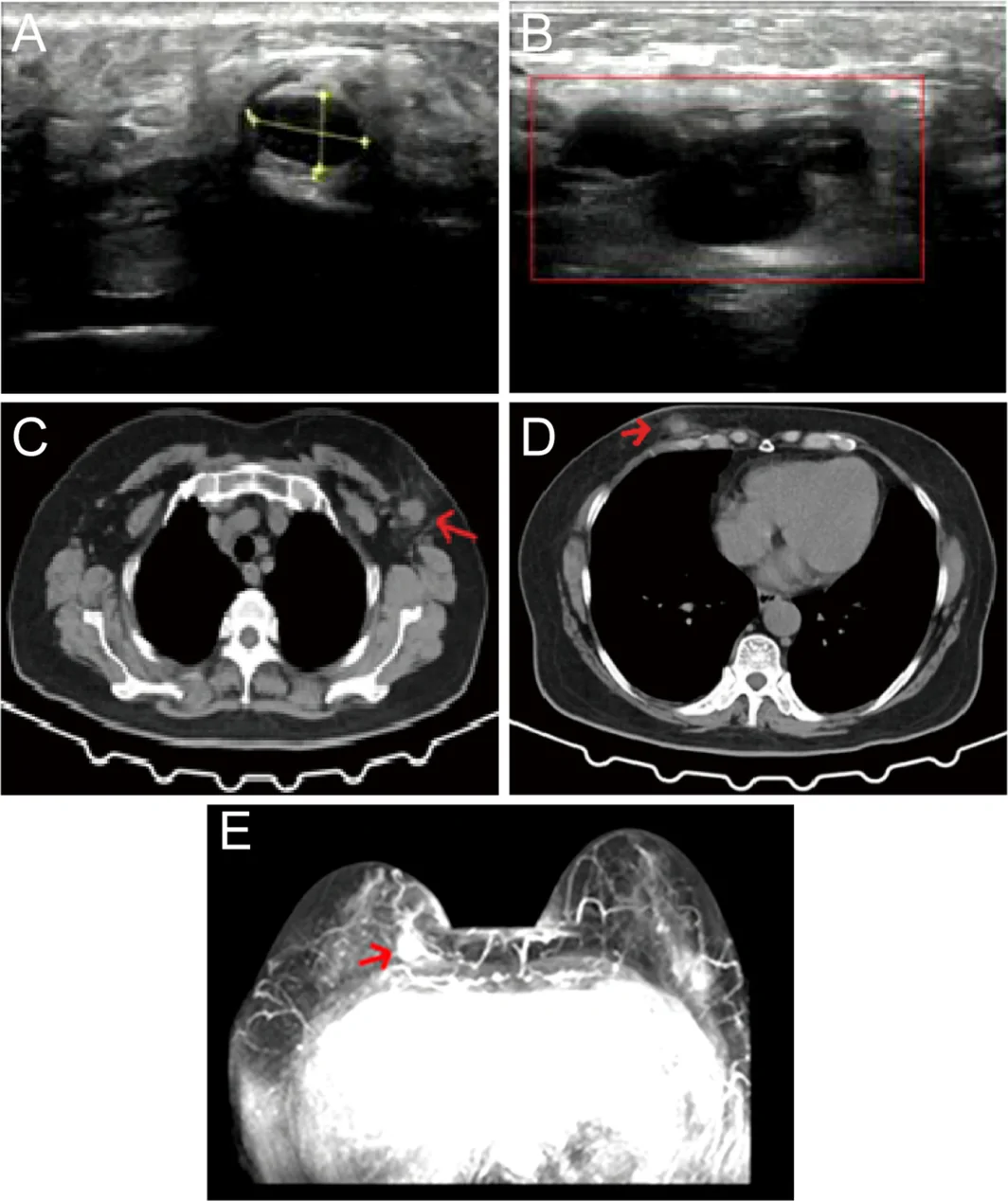
Multimodal imaging of the right breast lesion and left axillary lymph node involvement. (A) Breast ultrasound showing a mixed‐echogenic nodule at the 5 o'clock position of the right breast. (B) Ultrasound of the left axilla showing an enlarged lymph node suspicious for metastasis. (C) Contrast‐enhanced CT of the chest showing enlarged lymph nodes in the left axilla. (D) Contrast‐enhanced CT of the chest showing a nodule in the inner lower quadrant of the right breast. (E) Contrast‐enhanced breast MRI showing an enhancing lesion in the right breast.

On 20 February 2024, laboratory tests revealed an elevated serum cancer antigen 125 level of 52.73 U/mL. Breast ultrasonography showed, at the glandular margin in the 5 o'clock position of the right breast, a mixed‐echogenic lesion measuring approximately 10.7 × 6.5 × 11.3 mm with clear margins and a regular shape; multiple punctate hyperechoic foci with a maximum diameter of about 0.7 mm were noted within the lesion, and color Doppler flow imaging detected no obvious internal blood flow (Figure 1A). In the left axilla, an irregular, heterogeneous hypoechoic mass measuring approximately 31 × 11 × 22 mm was seen, with indistinct borders and inhomogeneous internal echoes; multiple strong echoes up to 1.4 mm in diameter were visible within the lesion. Color Doppler flow imaging demonstrated abundant peripheral vascularity, with increased echogenicity of the surrounding soft tissue. A tortuous tubular echo, about 2.6 mm in diameter with poor acoustic transmission and low internal echoes, was observed, which appeared continuous with the heterogeneous hypoechoic axillary mass; no obvious blood flow was detected within this tubular structure (Figure 1B).

Non‐contrast and contrast‐enhanced computed tomography (CT) of the chest on 20 February 2024 showed multiple enlarged lymph nodes in the left axilla and a nodule in the lower inner quadrant of the right breast. Breast magnetic resonance imaging showed an approximately 11 × 8‐mm lesion in the lower inner quadrant of the right breast and enlarged left axillary lymph nodes (Figure 1C–E).

**FIGURE 2:**
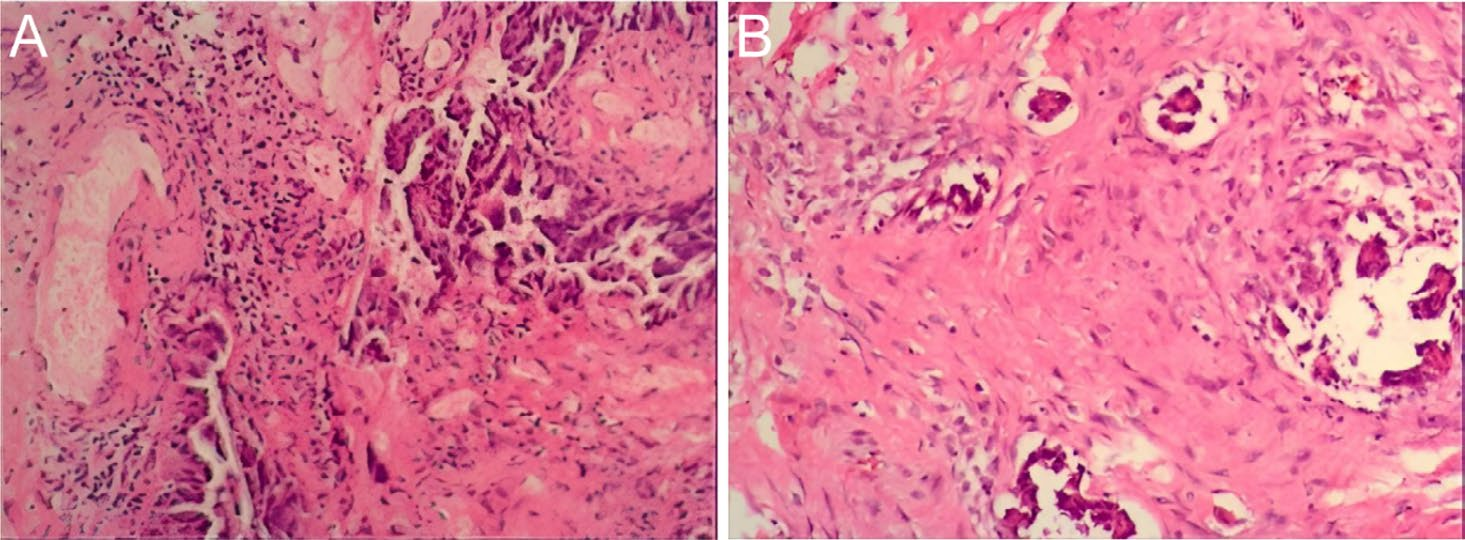
Histopathology of the primary ovarian tumor. (A and B) Microscopic examination (H&E ×100) revealed a serous borderline ovarian tumor with focal microinvasion.

**FIGURE 3:**
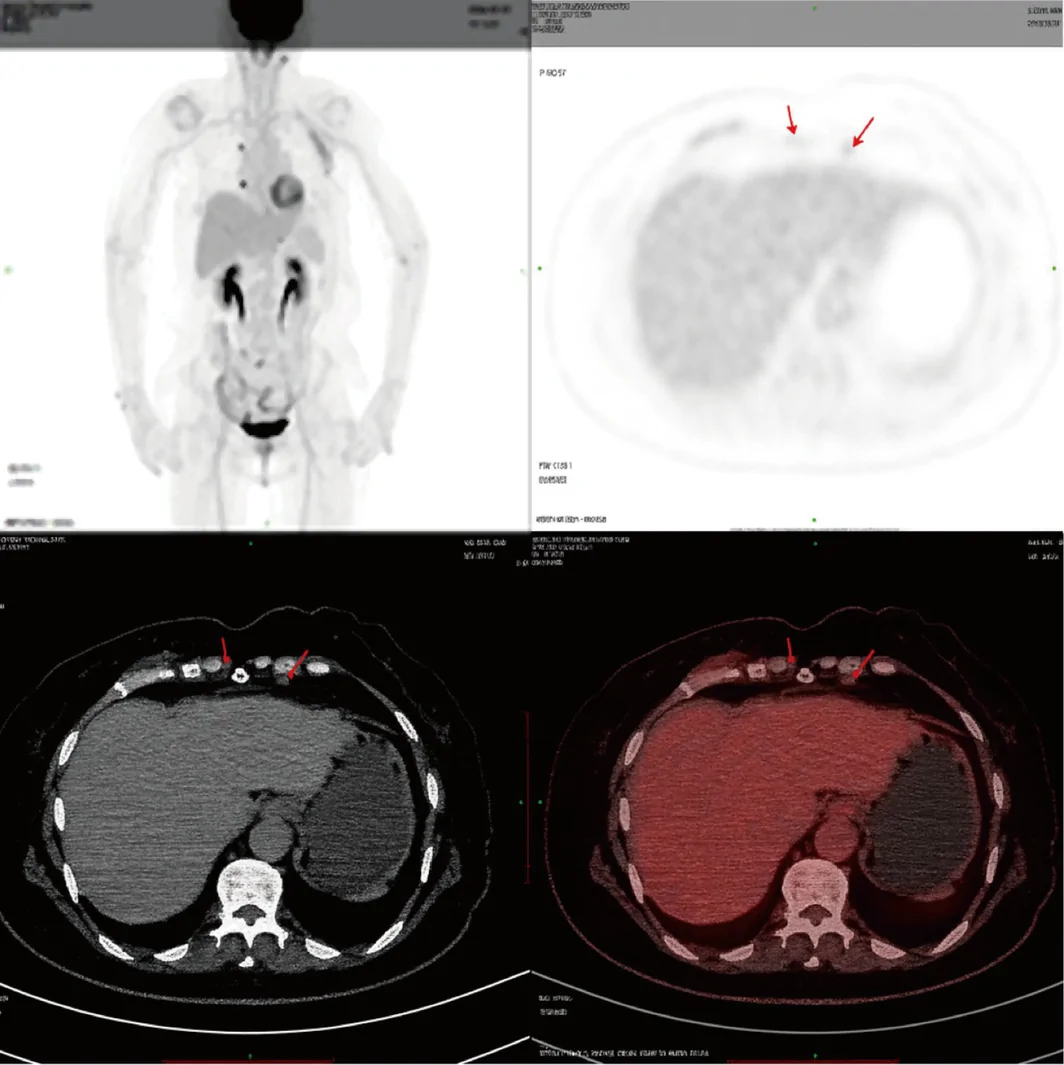
PET‐CT demonstrating disseminated nodal metastases of ovarian tumor.

Given the coexistence of a right breast nodule and contralateral axillary lymphadenopathy in a patient with a recent history of ovarian carcinoma, the principal differential diagnoses included a new primary breast carcinoma with axillary metastases versus metastatic ovarian carcinoma involving the breast and axillary nodes. Based on this diagnostic uncertainty, the patient's family was advised to bring the ovarian cancer surgical specimens to our hospital for expert pathologic re‐evaluation, and a PET‐CT examination was arranged. Expert pathologic re‐evaluation of the left adnexa and cystic content on 6 March 2024 confirmed a serous borderline papillary cystadenoma with focal invasion (well‐differentiated serous cystadenocarcinoma) (Figure 2A,B). PET‐CT performed on 4 March 2024 revealed multiple hypermetabolic lymph nodes along both sides of the sternum, in the right 11th–12th intercostal spaces, at the left anterior phrenic angle and diaphragmatic crura, and posterior to the pancreas, highly suggestive of nodal metastases (Figure 3).

To further clarify the nature of the lesions, image‐guided biopsies were performed on 20 February 2024. Core biopsy of the right breast mass showed large areas of necrotic tissue with a few residual enlarged nuclear cell fragments, raising concern for tumor necrosis. Fine‐needle aspiration of the left axillary lymph node revealed metastatic carcinoma in lymphoid tissue. The immunoprofile partially supported, but did not definitively confirm, a breast primary. Immunohistochemistry showed CK (AE1/AE3) (+), PR (5%, weak +), ER (70%+), GATA‐3 (−), HER‐2 (1+), and Ki‐67 (80%+).

**FIGURE 4:**
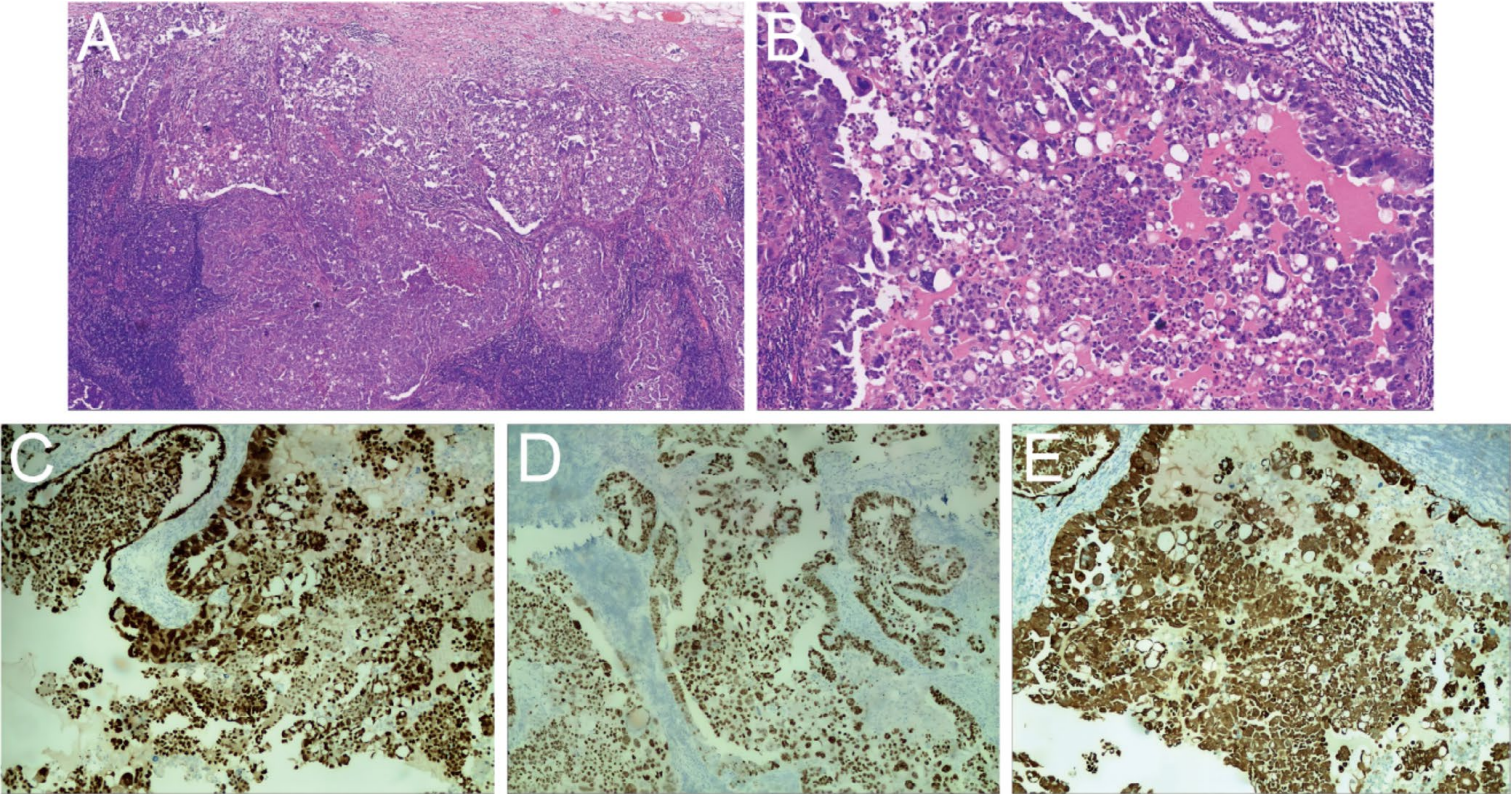
Histopathology and immunophenotype of the left axillary lymph node metastases. (A) Hematoxylin and eosin staining of the left axillary lymph node showing features consistent with high‐grade serous carcinoma (×40). (B) Higher‐magnification H&E section of the left axillary lymph node confirming high‐grade serous carcinoma (×100). (C) Immunohistochemical staining for PAX‐8 showing diffuse nuclear positivity in the metastatic carcinoma cells of the left axillary lymph node (×100). (D) Immunohistochemical staining for WT‐1 demonstrating nuclear positivity in the metastatic carcinoma cells of the left axillary lymph node (×100). (E) Immunohistochemical staining for P16 showing strong, diffuse nuclear and cytoplasmic positivity in the metastatic carcinoma cells of the left axillary lymph node (×100).

The patient subsequently underwent left axillary lymph node dissection combined with excision of the right breast mass on 26 February 2024. Routine postoperative histopathology of the right breast specimen, which was entirely submitted for examination, demonstrated extensive coagulative necrosis with a small focus of carcinoma cell clusters (approximately 100 cells) at the periphery; the morphology was consistent with invasive/metastatic carcinoma. Immunohistochemistry showed PAX‐8 (+), WT‐1 (+), P16 (diffuse +), P53 (completely negative, mutant‐type pattern), GATA‐3 (−), TRPS1 (+), and Ki‐67 (60%+). Among the 15 dissected left axillary lymph nodes, 2 harbored macrometastases, morphologically consistent with high‐grade serous carcinoma of ovarian origin (Figure 4A,B). Immunohistochemistry demonstrated CK7 (+), PAX‐8 (+), WT‐1 (+), TRPS1 (+), GATA‐3 (−), GCDFP‐15 (−), Ki‐67 (60%+), ER (80%, moderate +), PR (−), and HER‐2 (0) (Figure 4C–E). The breast lesion was therefore not staged as a primary breast carcinoma; according to the available records, the overall findings supported recurrent metastatic ovarian‐origin carcinoma involving the right breast and left axillary lymph nodes. Genetic testing showed no pathogenic mutations in BRCA1 or BRCA2.

## 4 Conclusion and Results (Outcome and Follow‐Up)

**FIGURE 5:**
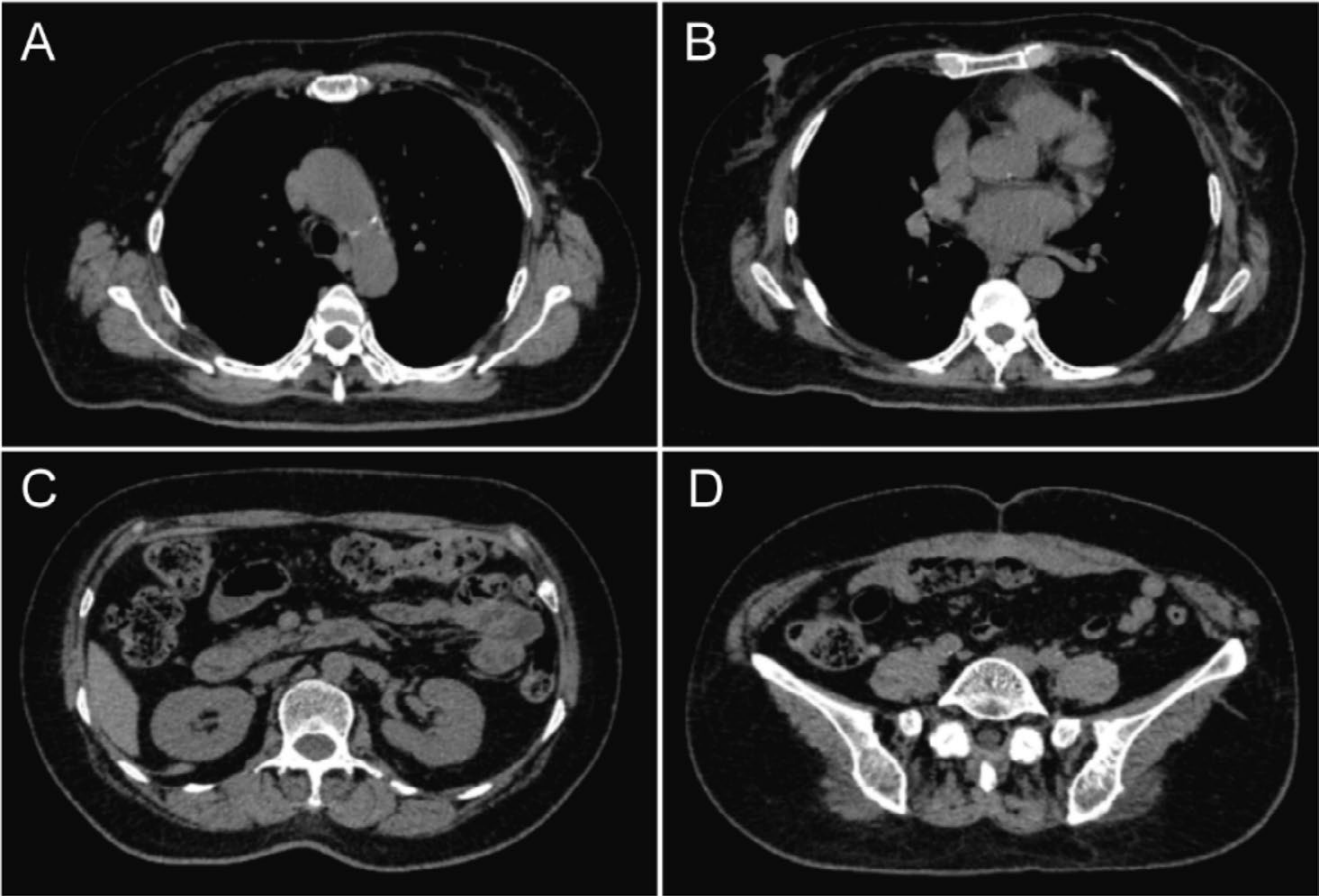
Follow‐up CT images showing no obvious enlarged lymph nodes or radiologic evidence of disease progression. (A) Chest CT showing no obvious enlarged parasternal lymph nodes. (B) Chest CT showing no obvious recurrent breast lesion or axillary lymphadenopathy. (C) Abdominal CT showing no obvious enlarged abdominal or retroperitoneal lymph nodes. (D) Pelvic CT showing no obvious enlarged pelvic lymph nodes.

Following surgery, the patient was transferred to the gynecology department and received six cycles of systemic chemotherapy with paclitaxel plus carboplatin, with the first three cycles administered on 22 March, 12 April, and 8 May 2024, followed by maintenance therapy with niraparib tosylate capsules at 200 mg orally once daily. At the most recent follow‐up on March 13, 2025, follow‐up CT showed no obvious enlarged mediastinal, abdominal, retroperitoneal, or pelvic lymph nodes, and no ascites. Serum tumor markers were within normal limits, including cancer antigen 125 (10.74 U/mL), carbohydrate antigen 19–9 (0.25 U/mL), and carcinoembryonic antigen (2.08 ng/mL), indicating no radiologic evidence of disease progression (Figure 5).

The available records documented an uneventful chemotherapy course without major adverse events. Supportive liver‐protective, gastric‐protective, and antiemetic treatments were administered as needed.

During subsequent follow‐up, the available records documented preserved oral intake, normal bowel and urinary function, and no obvious weight change. Together with the imaging and tumor marker results, these findings indicated a favorable short‐term clinical and radiologic outcome.

## 5 Discussion

Although primary breast cancer is common, metastases to the breast from extramammary primaries account for only 0.2%–1.3% of all malignant breast tumors [3]. Among these, patients who initially present with a breast mass but are ultimately diagnosed with metastatic ovarian carcinoma represent as few as 0.03%–0.6% of breast malignancies [4, 5, 6]. Such presentations are therefore highly prone to misdiagnosis as primary breast cancer in routine clinical practice [7]. In addition, distant metastasis is relatively uncommon in serous borderline ovarian tumors with focal microinvasion, and this patient had previously undergone pelvic lymph node dissection with no nodal involvement on postoperative pathology. The subsequent development of distant metastases to the breast and contralateral axillary lymph nodes, accompanied by a marked increase in tumor grade, makes this case clinically instructive.

In this patient, core needle biopsy of the breast lesion was nondiagnostic, whereas fine‐needle aspiration of the contralateral axillary lymph node confirmed metastatic carcinoma. Because most breast lymphatic drainage goes to the ipsilateral axillary nodes, the ipsilateral breast is usually considered the most likely primary site. In this case, however, the axillary immunoprofile only partially supported a breast origin. The right breast lesion was considered more likely to represent a parenchymal breast metastasis rather than an intramammary lymph node metastasis. This interpretation was based on the imaging localization within the breast and the postoperative pathologic finding that the excised specimen was consistent with invasive/metastatic carcinoma of ovarian origin. However, because the lesion showed extensive coagulative necrosis with only a small residual focus of viable carcinoma, this distinction could not be made with absolute certainty. If the tumor were primary breast cancer, a key question would be whether it arose from the ipsilateral or contralateral breast; in either case, occult breast cancer remained a diagnostic consideration. Because core biopsy of the right breast lesion was nondiagnostic and fine‐needle aspiration of the contralateral axillary lymph node confirmed metastatic carcinoma without identifying the primary site, additional tissue was needed for a definitive diagnosis. After multidisciplinary discussion involving breast surgery, gynecologic oncology, radiology, and pathology, surgical tissue acquisition was performed. In retrospect, excisional biopsy of the most suspicious axillary lymph node might have provided sufficient diagnostic tissue with less invasiveness. Therefore, this case highlights that axillary lymph node dissection should not be considered a routine diagnostic approach in similar cases; the least invasive procedure capable of providing adequate tissue should be prioritized. Given the patient's prior history of ovarian carcinoma, additional ovary‐specific immunohistochemical markers were applied to further clarify the origin. PAX8 and WT1 supported Müllerian/ovarian origin, whereas GATA3 and GCDFP‐15 are commonly used to support a breast primary [8, 9, 10, 11, 12, 13]. Importantly, ER and TRPS1 positivity did not establish breast origin in isolation, because ER expression is common in serous ovarian carcinoma and TRPS1 expression has also been reported in gynecologic carcinomas [14, 15]. In this case, the final immunohistochemical findings confirmed that both the right breast mass and the left axillary lymph node metastasis were consistent with ovarian carcinoma.

The route by which ovarian carcinoma spreads to the breast and axillary lymph nodes remains incompletely elucidated. One possible route is dissemination through the para‐aortic nodes or peritoneal cavity, followed by diaphragmatic lymphatic spread to the right breast. Tumor cells may then migrate via the internal mammary lymph nodes, deep lymphatic channels beneath the thoracic fascia, or the subcutaneous lymphatic plexus to the contralateral internal mammary chain, and finally reach the left axillary lymph nodes [16]. Lee et al. further reported the existence of preferential lymphatic stations, such as the thoracic duct and left supraclavicular lymph nodes, which may increase the risk of ovarian carcinoma metastasizing to the left breast [17]. The uniqueness of our case lies in the fact that pelvic lymph node dissection and omentectomy at the time of ovarian cancer surgery showed no nodal or omental tumor involvement, yet the patient subsequently developed metastases to the right breast and contralateral axilla. The absence of standardized adjuvant chemotherapy after the initial ovarian cancer surgery may have contributed to this pattern of dissemination, although a causal relationship cannot be inferred from a single case.

The discrepancy between the primary ovarian tumor and the metastatic axillary lesion requires cautious interpretation. Postoperative pathology of the ovarian tumor in this patient demonstrated a serous borderline ovarian tumor with focal microinvasion, suggesting relatively low malignant potential and an extremely low probability of breast metastasis. By contrast, the axillary lymph node lesion was diagnosed as high‐grade serous carcinoma. This discrepancy may reflect limitations of the initial pathologic diagnosis, sampling error, intratumoral heterogeneity, progression from a low‐grade/borderline lesion to a high‐grade tumor, or additional genetic or epigenetic alterations during distant dissemination [18, 19]. Although the mechanism cannot be established from a single case, the marked difference in grade highlights the need for pathologic re‐evaluation and integrated clinicopathologic interpretation when metastatic disease is suspected. Furthermore, previous studies have shown that patients with ovarian carcinoma metastasizing to the breast have a dismal prognosis: reported survival times after the diagnosis of breast metastasis range from 13 days to 3.5 years, with a 1‐year survival rate of less than 50% [20]. These observations underscore the need for timely systemic therapy and careful follow‐up in patients with advanced metastatic ovarian carcinoma.

This report has several limitations. The initial ovarian operation was performed at an outside hospital, and the available records did not include detailed postoperative performance‐status scores or laboratory values explaining why adjuvant chemotherapy was deferred after the first operation. Nevertheless, the diagnostic conclusion was supported by expert pathologic re‐evaluation and a concordant immunohistochemical profile across the breast and axillary lesions.

In summary, we report a rare case of breast metastasis with contralateral axillary lymph node involvement occurring after surgery for a serous borderline ovarian tumor with focal microinvasion. In patients with a previous malignancy, atypical breast or axillary lesions should be evaluated for both primary breast cancer and metastasis from the earlier tumor. Thorough history taking, multidisciplinary evaluation, targeted immunohistochemistry, and an appropriately conservative tissue‐sampling strategy are essential for accurate differential diagnosis.

## Author Contributions

**Sihan Zhang:** conceptualization, investigation, supervision, writing – original draft, writing – review and editing. **Zhiyuan Zhang:** conceptualization, investigation, supervision, writing – original draft, writing – review and editing. **Jiaru Gao:** data curation, investigation. **Mengshen Wang:** data curation, investigation. **Minghui Zheng:** data curation, investigation. **Qiaonan Ye:** data curation, investigation. **Chenxi Yuan:** data curation, investigation. **Linjiao Jia:** project administration, writing – review and editing. **Wentao Li:** funding acquisition, project administration, writing – review and editing.

## Funding

This work was supported by the Henan Provincial Key Scientific and Technological Project (Grant No. 252102311054), Department of Education of Henan Province. This work was supported by the Key Project of the Henan Province Medical Science and Technology Research Plan Jointly Constructed by the Provincial and Ministerial Departments (SBGJ202502016, SBGJ202502017).

## Ethics Statement

The study was approved by the Ethics Committee of Zhengzhou University People's Hospital (approval number: hnsrmyy2020‐11). Written informed consent for publication was obtained from the patient or her legal representative.

## Consent

We confirm that the manuscript has been read and approved by all named authors, and that there are no other persons who satisfied the criteria for authorship but are not listed. We also confirm that the order of the authors listed in the manuscript has been approved by all of us.

## Conflicts of Interest

The authors declare no conflicts of interest.

## Data Availability

No datasets were generated or analyzed during the current study.
